# Anti-CD20-Mediated B Cell Depletion Is Associated With Bone Preservation in Lymphoma Patients and Bone Mass Increase in Mice

**DOI:** 10.3389/fimmu.2020.561294

**Published:** 2020-10-19

**Authors:** Albert Kolomansky, Irit Kaye, Nathalie Ben-Califa, Anton Gorodov, Zamzam Awida, Ofer Sadovnic, Maria Ibrahim, Tamar Liron, Sahar Hiram-Bab, Howard S. Oster, Nadav Sarid, Chava Perry, Yankel Gabet, Moshe Mittelman, Drorit Neumann

**Affiliations:** ^1^Department of Cell and Developmental Biology, Tel Aviv University, Tel Aviv, Israel; ^2^Department of Medicine A, Tel Aviv Sourasky Medical Center, Tel Aviv, Israel; ^3^Sackler Faculty of Medicine, Tel Aviv University, Tel Aviv, Israel; ^4^Open University of Israel, Ra'anana, Israel; ^5^Department of Radiology, Tel Aviv Sourasky Medical Center, Tel Aviv, Israel; ^6^Department of Anatomy and Anthropology, Tel Aviv University, Tel Aviv, Israel; ^7^Tel Aviv Sourasky Medical Center, Institute of Hematology, Tel Aviv, Israel

**Keywords:** rituximab, follicular lymphoma, anti-CD20 antibodies, RANKL (receptor activator for nuclear factor k B ligand), B cell depletion, bone density, CD115

## Abstract

Immunotherapy with anti-CD20-specific antibodies (rituximab), has become the standard of care for B cell lymphoproliferative disorders and many autoimmune diseases. In rheumatological patients the effect of rituximab on bone mass yielded conflicting results, while in lymphoma patients it has not yet been described. Here, we used cross-sectional X-ray imaging (CT/PET-CT) to serially assess bone density in patients with follicular lymphoma receiving rituximab maintenance therapy. Remarkably, this treatment prevented the decline in bone mass observed in the control group of patients who did not receive active maintenance therapy. In accordance with these data, anti-CD20-mediated B cell depletion in normal C57BL/6J female mice led to a significant increase in bone mass, as reflected by a 7.7% increase in bone mineral density (whole femur), and a ~5% increase in cortical as well as trabecular tissue mineral density. Administration of anti-CD20 antibodies resulted in a significant decrease in osteoclastogenic signals, including RANKL, which correlated with a reduction in osteoclastogenic potential of bone marrow cells derived from B-cell-depleted animals. Taken together, our data suggest that in addition to its anti-tumor activity, anti-CD20 treatment has a favorable effect on bone mass. Our murine studies indicate that B cell depletion has a direct effect on bone remodeling.

## Introduction

Immunotherapy with anti-CD20-specific antibodies has become the standard of care for many hematological and autoimmune diseases (1). Current treatment protocols for virtually all B cell lymphoproliferative neoplasms include anti-CD20-directed therapy (anti-CD20), with rituximab being the first-in-class drug. Recently, third generation anti-CD20 antibodies have been developed and introduced into clinical practice (1).

Despite a significant amount of published research linking B cells to bone homeostasis (2–5), only a few studies have addressed the effect of anti-CD20 on bone mass in human patients, and no studies have yet addressed this issue in hematological patients. Studies evaluating the skeletal effects of rituximab in rheumatological patients reported conflicting results. One study on rituximab treatment in patients with rheumatoid arthritis (RA), described an increase in lumbar spine BMD (bone mineral density) irrespective of clinical response, whereas in the femur, rituximab merely prevented bone loss, and only in responders (6). Another study suggested that treatment with rituximab reduced osteoclast activity as assessed by serum markers of bone resorption (7), and a recent study, also in RA patients (8), demonstrated a significant reduction in the femoral BMD with no effect in the spine. A similar effect was found in patients with systemic lupus erythematosus (female patients only), especially in those who attained a good clinical response to the treatment (9).

B lymphocytes can regulate bone metabolism *via* their ability to secrete receptor activator for nuclear factor κ B ligand (RANKL) and osteoprotegerin (OPG) (3, 10, 11). Depending on their state and/or mode of activation, B cells were shown to inhibit (12) or enhance (13–15) osteoclastogenesis by these signals. For example, deletion of RANKL in B cells prevented ovariectomy (OVX)-induced trabecular bone loss (11). However, constitutive depletion of IgM^+^ B cells in mice (i.e., both immature and mature B cells) led to a reduction in bone mass (10). An inverse relationship also exists as bone cells are involved in B cell commitment, development, and maturation (16–19). Lastly, in spite of the fact that osteoclasts (OCs) and B cells arise from distinct committed progenitors, several lines of evidence suggest that B cells can contribute directly to bone remodeling by transdifferentiating into bone-resorbing OCs *in vitro* (5, 10, 20, 21). B cells were shown to undergo trans-differentiation to macrophages (22) and these cells share similarities with osteoclast precursors (22). In this context, we have recently demonstrated B-cell-derived osteoclastogenesis both *in vitro* and *in vivo* (23). Notably, our data indicate that among BM B cells, only Pro-B cells bearing the receptor for macrophage colony-stimulating factor (MCSF-R, also known as cFms or CD115), are capable of giving rise to functional osteoclasts (23). Here, we evaluated the skeletal effects of anti-CD20 antibodies in a cohort of hematological patients with follicular lymphoma and found that this treatment is associated with a bone-preserving effect. Consistent with this clinical data, administration of anti-CD20 antibodies in a murine model resulted in a decrease in osteoclastogenic signals as well as osteoclastogenic potential *in vitro*.

## Methods

### Patients

We retrospectively evaluated the skeletal effects of rituximab (anti-CD20 antibody) in patients with low-grade (follicular) lymphoma (FL), who were treated with rituximab in the setting of maintenance therapy, that is, a period when rituximab was administered periodically, as monotherapy. This approach was chosen to avoid the potential bias of concurrent treatments that may affect bone metabolism (e.g., corticosteroids). The treatment group included patients with low-grade FL who received rituximab maintenance following rituximab-containing chemo-immunotherapy induction with one of the standard protocols used for this indication in the relevant time period (24). The control group consisted of patients with low-grade lymphoma who did not receive rituximab maintenance or any other treatment, following rituximab-containing chemo-immunotherapy induction. These included patients with FL who received treatment prior to the inclusion of maintenance therapy in the treatment schedule, and patients diagnosed with low-grade lymphoma other than FL (e.g., marginal zone lymphoma). Only patients with at least two consecutive CT-based cross-sectional imaging studies performed at the Tel Aviv Sourasky Medical Center (TASMC) during the first year post-induction, were included in the analysis. Patients with chronic kidney disease or active non-hematological malignancies, other than squamous or basal cell carcinoma of the skin, were excluded. The study was approved by the Sourasky Medical Center institutional ethical committee, permit number 0418-18-TLV.

### Evaluation of Bone Mass in Patients

**Figure 1:**
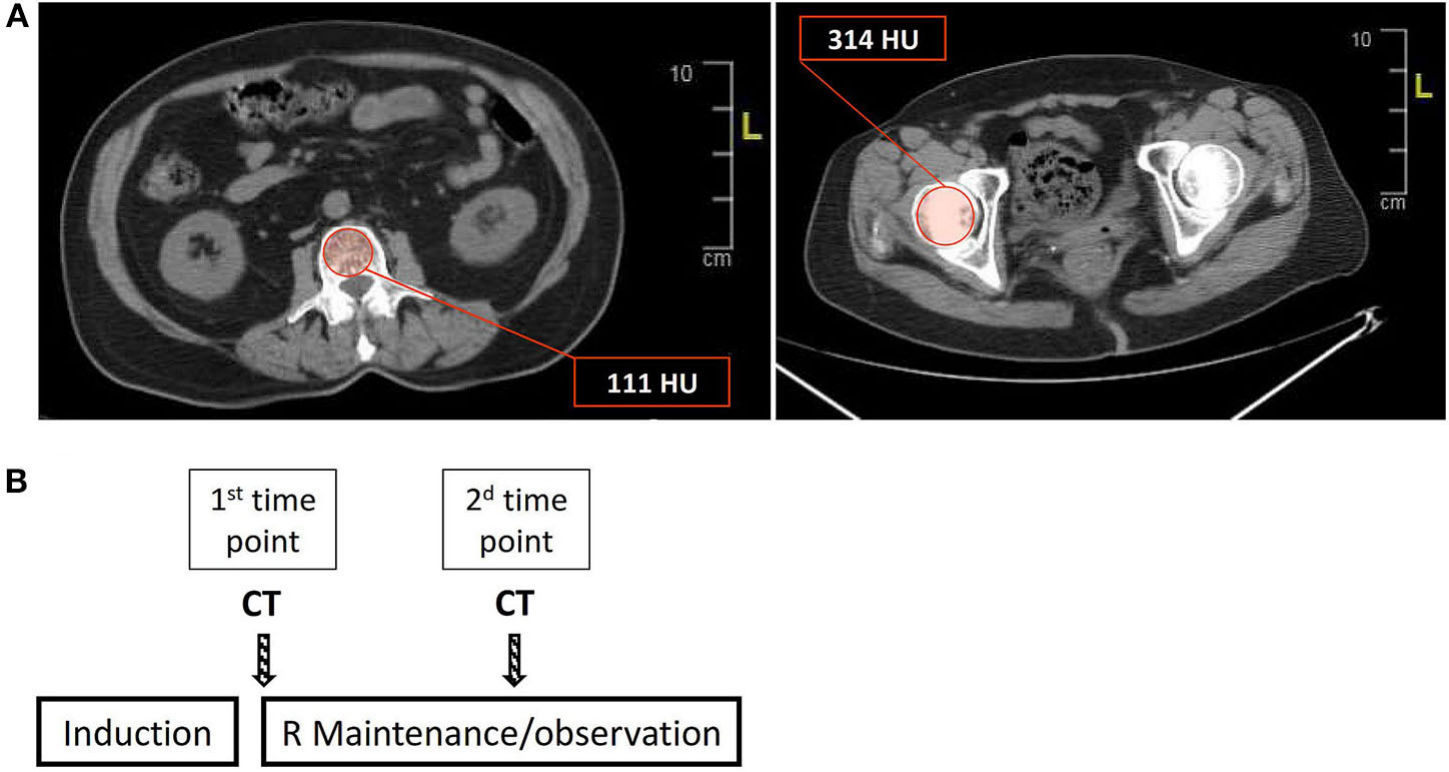
Estimation of bone mass using cross-sectional CT-based imaging. **(A)** Bone mineral density (BMD) was measured in the lumbar spine (L1–L4) and femoral head, using regions of interest (ROIs) as depicted (red circle) in the left and right panels for the lumbar vertebrae (L1 in this section) and femoral head, respectively. The values in the red boxes reflect the average of the HU (Hounsfield units) values in the ROI (see also “Methods” for a detailed description of BMD calculation). **(B)** A scheme describing the timing of the sequential BMD measurements, that is, the timing of the CT/PET scans in relation to the treatment steps. R = rituximab.

Based on recent reports indicating a high degree of correlation between CT-based cross-sectional imaging and DEXA scans (25, 26), we utilized available PET-CT or conventional CT scans to evaluate bone mass in the lumbar spine and femoral head in the study population. In the lumbar spine, we used axial sections in the central portion of the vertebrae to select a region of interest (ROI) along the inner circumference of the vertebral body as demonstrated in Figure 1A (left panel). Bone mineral density (BMD) in each section was determined from the average of the signal intensities measured in the ROI and expressed in Hounsfield units (HU). BMD of the lumbar spine was determined by averaging the values obtained from BMD measurements in the vertebrae L1 through L4. BMD in the femoral head was determined by averaging the BMD values obtained from 4 to 5 axial sections through the femoral head (Figure 1A, right panel). For each patient, two consecutive measurements of bone mass were performed: first (“1^st^ time point”)—immediately after completing induction (as part of the assessment of response to treatment), and second—at the time of next evaluation of disease status, usually 6 months later (designated as “2^nd^ time point,” Figure 1B).

### Experimental Animals and Administration of Anti-CD20

Mouse handling and all experimental procedures were approved by the Institutional Animal Care and Use Committee of the Tel-Aviv University (permit number: 01-016-010). Female C57BL/6J wild-type mice (7–10 weeks, substrain RccHsd, Envigo, Israel) were used in the study. Mouse anti-mouse CD20 antibodies (anti-mCD20), kindly provided by Genentech, were used to deplete mouse CD20^+^ B cells. Two equal doses of anti-mCD20 (100 μg/dose) were administered 2 weeks apart (on days 1 and 14), with the first dose administered intravenously (IV) through the tail vein and the second intraperitoneally (IP). Mice were sacrificed 2 to 3 weeks after the second dose giving a total treatment period of 4 to 5 weeks.

### Assessment of Bone Mass in Experimental Animals Using Micro-Computed Tomography (μCT)

Femora (one per mouse) were examined using the μCT50 system (Scanco Medical AG, Switzerland) (27, 28). Briefly, scans were performed at a 10 μm resolution. The mineralized tissues were segmented by a global thresholding procedure (29). Trabecular bone parameters were measured in the secondary spongiosa of the distal femoral metaphysis, which was further divided into proximal and distal halves. Cortical parameters were determined in a 1 mm height ring in the mid-diaphyseal region. Volumetric bone mineral density (vBMD) was calculated by the proprietary Scanco software with reference to a calibrated phantom, and expressed as mg hydroxyapatite per cm^3^ of tissue (mgHA/cm^3^).

### Osteoclast Differentiation *in vitro*

Isolated bone marrow cells were seeded on tissue culture-treated 96-well plates in α-MEM with 10% FBS, M-CSF [in the form of 2% *v*/*v* culture supernatant from CMG 14–12 cells, containing 1.3 μg/ml M-CSF (22, 30)], and 50 ng/ml recombinant murine RANKL (R&D Systems, Minneapolis, MN). Culture medium was replaced every 2–3 days. After 4–5 days, the cells were fixed and stained for tartrate-resistant acid phosphatase (TRAP, Sigma-Aldrich, MO, USA). Osteoclast surface area was measured using ImageJ software (NIH, Bethesda, MD).

### Flow Cytometry

Bone marrow (BM) cells were flushed from femurs or tibias, and red blood cells were lysed using ACK lysis buffer (Quality Biological, Gaithersburg, MD). The cells were then stained for 30 min at 4°C with conjugated anti-mouse antibodies (see Supplementary Table 1 for the list of the antibodies used). After this time, cells were washed with PBS containing 2% FBS and analyzed by either Gallios or Cytoflex flow cytometers and Kaluza software (all from Beckman Coulter, Indianapolis, USA).

### Real-Time Quantitative PCR

Total RNA was extracted from flushed BM cells using TriRNA Pure kit (Cat.# TRPD200, Geneaid, New Taipei city, Taiwan). cDNA was synthesized using the qScript cDNA synthesis kit (Quantabio, Massachusetts, USA). Bone specimens were first homogenized using a mechanical homogenizer, followed by RNA extraction and cDNA synthesis as described for BM cells. “Real-time” quantitative PCR (RQ-PCR) was performed on a StepOnePlus instrument using SYBR Green reagent (both from Applied Biosystems, California, USA). Relative gene expression was calculated using the ΔΔCT method following normalization to the expression of HPRT as a housekeeping gene. All RQ-PCR experiments were performed in triplicate.

### Statistical Analysis

Categorical variables were compared using Fisher's exact test. Continuous variables were compared using either Wilcoxon matched-pairs signed rank test (BMD data of lymphoma patients) or unpaired Student's *t*-test (all others). Values were expressed as mean ± SEM (standard error of the mean). Statistical analysis was performed using GraphPad Prism 7 (San Diego CA, USA). Statistical significance was defined as *p* < 0.05.

## Results

### Treatment With Anti-CD20 Preserves Bone Mass in Patients With Follicular Lymphoma

**Figure 2:**
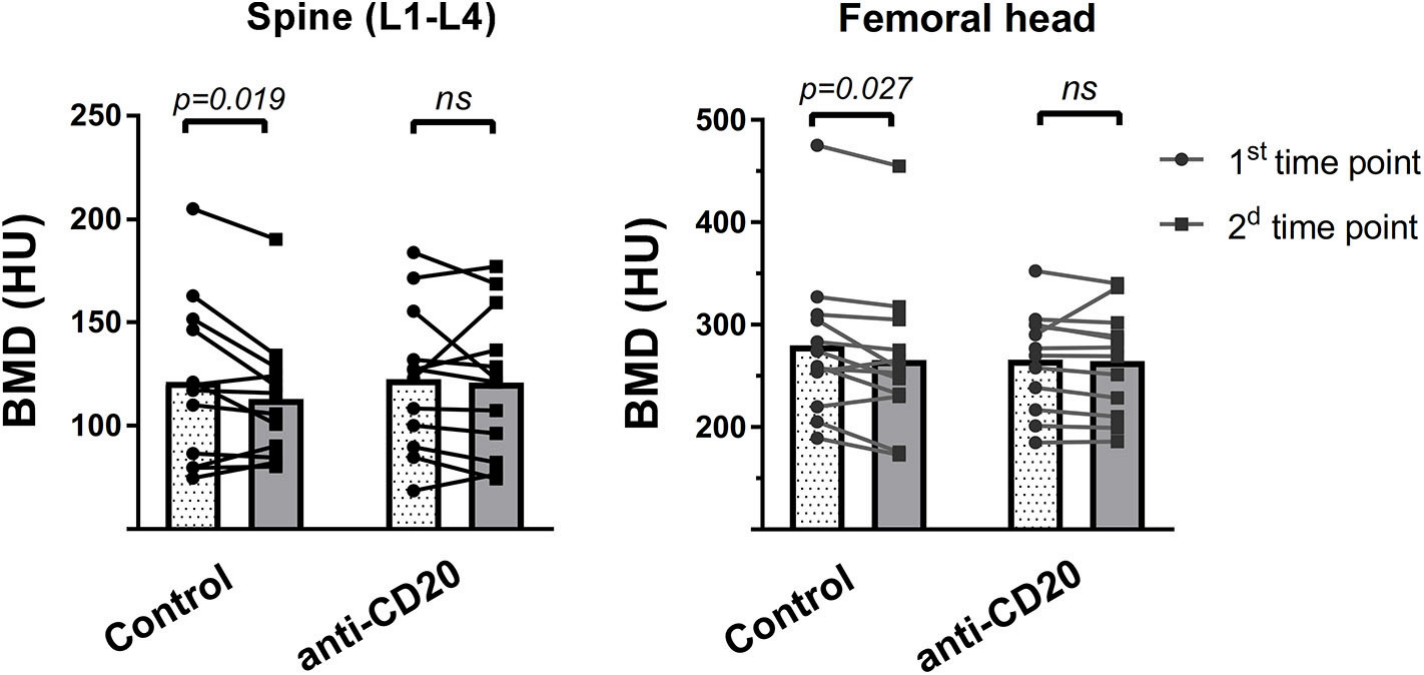
Rituximab maintenance treatment prevents post-induction bone loss. Two consecutive measurements of bone mass in the lumbar spine (left) and femoral head (right), as assessed using CT-based cross-sectional imaging, in patients with low grade lymphoma who received rituximab (R, anti-CD20) maintenance vs. those without R maintenance. Imaging studies (mostly PET-CT) were performed immediately after induction therapy (first time point), and then 6 to 7 months post-induction (second time point). Bar graphs represent corresponding means, whereas dots interconnected by lines denote individual pairs of consecutive measurements for every patient. *p-*values were calculated using Wilcoxon matched-pairs signed rank test. ns = not significant.

After reviewing the clinical charts of 125 patients with low-grade lymphoma followed up in the hematology department of TASMC between 2008 and 2016, we identified 24 patients [(12 in each group (treatment and control)] for final analysis (see “Methods” for inclusion and exclusion criteria). Demographic and basic clinical characteristics of the patient cohort are presented in Table 1. The groups did not differ in age, gender, or frequency of glucocorticoid-containing induction regimens. The control group included some cases of marginal zone lymphoma. All patients responded to first line induction chemoimmunotherapy with slightly more complete responses (CR) in the control group (*p* > 0.05). Bone analysis using CT-based cross-sectional imaging at the first and second time points (end of induction and following 6–7 months of maintenance/observation, respectively) revealed no change in the bone mass of patients who received rituximab maintenance (between the 2 time points). In contrast, patients in the control group (observation only) had a significant reduction in the trabecular bone mass as assessed in the lumbar spine and femoral head (95%CI [91.2–145 vs. 85.5–130], and [232–327 vs. 219–312]), 9% reduction and 5.25% reduction, respectively (*p* < 0.05 for both sets of data, Figure 2). Our data therefore suggest that, at least in the current clinical setting, treatment with anti-CD20 is associated with preservation of bone mass.

**Table 1:** Baseline characteristics of the patient cohort.

	**Control**	**Anti-CD20**	***P*-value**
	**(*n* = 12)**	**(*n* = 12)**	
Age, mean (range)	67 (51–79)	61 (43–77)	ns
Gender (M/F)	6/6	4/8	ns
**Diagnosis**, ***n*** **(%)**			
FL	8 (67)	12 (100)	ns
MZL	4 (33)	0 (0)	ns
**Induction therapy**			
R-CHOP	5	6	
R-CVP	5	1	
RFC	0	2	
BR	2	1	
Other^*^	0	2	
**GC-containing regimen**	10	7	ns
**R-containing regimen**	12	12	
**Response to induction regimen**			
CR/PR	10/2	6/6	ns
**# of R cycles in the maintenance phase**			
3 cycles	NA	7	
1–2 cycles		2	
4–6 cycles		3	

*FL, follicular lymphoma; MZL, marginal zone lymphoma*.

*R-CHOP: rituximab, cyclophosphamide, adriamycin, vincristine, and prednisone*.

*R-CVP: rituximab, cyclophosphamide, vincristine, and prednisone*.

*R-FC: rituximab, fludarabine, and cyclophosphamide*.

*BR; bendamustine and rituximab*.

* *R monotherapy, Chlorambucil+R – one patient each, GC, glucocorticoid*.

*ns, not significant*.

### Treatment With Anti-CD20 Increases Bone Mass in Mice

**Figure 3:**
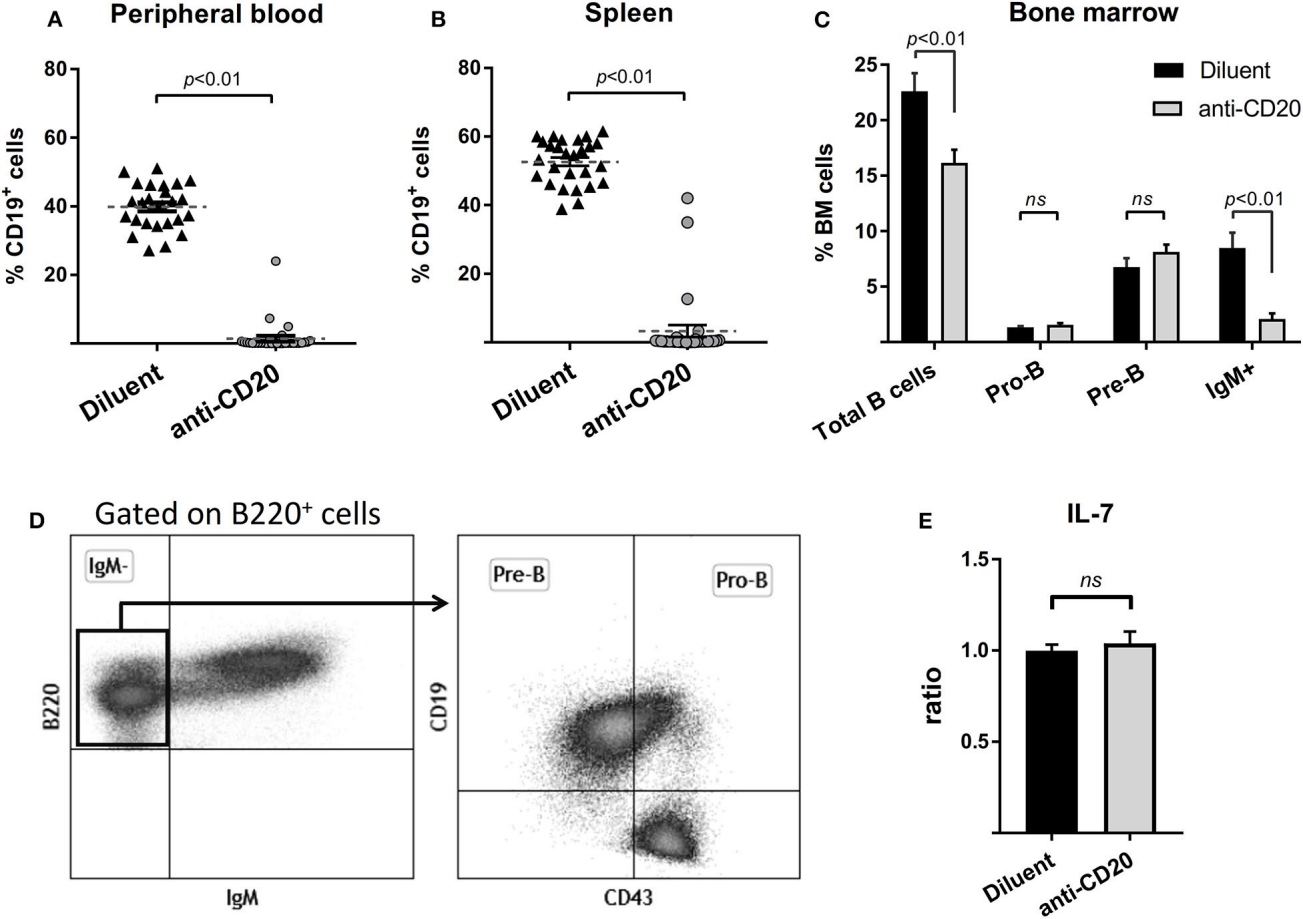
Administration of anti-mCD20 effectively depletes mature B cells in mice. The effect of anti-CD20 administration on the content of B cells in peripheral blood **(A)** and spleen **(B)** (defined by CD19 positivity). Summary of three separate experiments with a total of 27 diluent and 31 anti-CD20-treated female mice. Dashed lines with error bars **(A,B)** are mean ± SEM. **(C)** The effect of anti-CD20 on the distribution of B cell subpopulations in the bone marrow (BM) of mice described above. The gating strategy is presented in **(D)**, Values are mean ± SEM. **(E)** The effect of depletion of mature B cells on the expression of interleukin 7 (IL-7) as assessed by RQ-PCR on whole bone (proximal tibia). Values are mean ± SEM of ΔΔCT values normalized to the diluent group of each individual experiment (see above). ns = not significant.

To address the question of whether the preservation of bone mass observed in lymphoma patients treated with rituximab maintenance is due to bone-anabolic effects induced by prolonged B cell depletion, or possibly due to better control of the neoplastic process, we treated wild-type female mice with anti-mCD20 as described (see “methods”). The results presented in Figure 3, demonstrate a profound depletion of B cells from the peripheral blood and spleen (Figures 3A,B), as well as a 75% reduction in the IgM^+^ cell fraction in the BM (Figure 3C, see gating strategy in 3D) of the anti-CD20-treated animals compared to controls. Notably, there was no significant change in the Pro-B and Pre-B cell fractions, which is in line with the observation that administration of anti-mCD20 did not alter the expression of IL-7 in whole bone tissue (Figure 3E).

**Figure 4:**
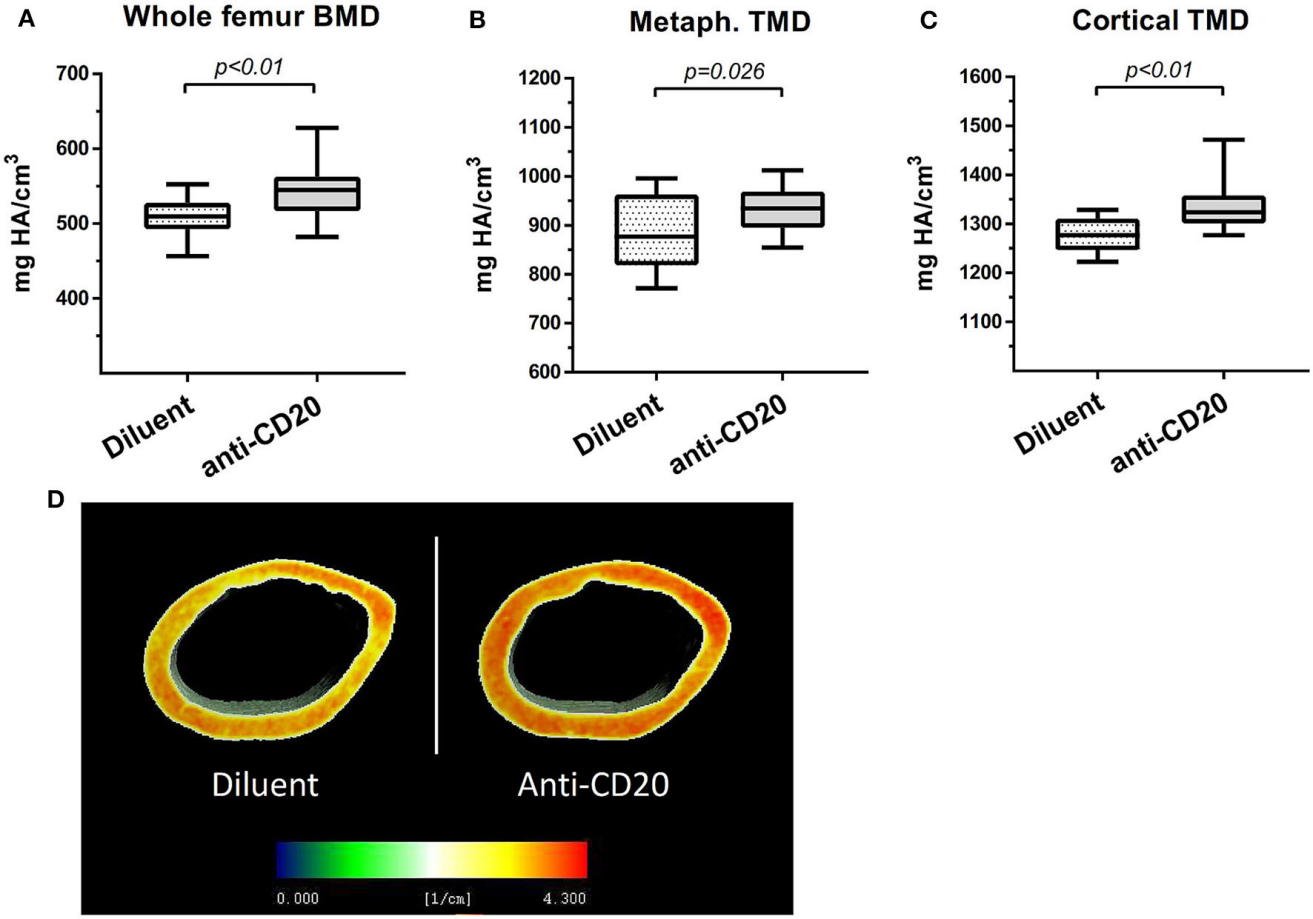
Depletion of mature B cells is associated with an increase in bone mass in mice. **(A)** Volumetric bone mineral density (vBMD) of the whole femur. **(B)** Metaphyseal tissue mineral density (TMD) and **(C)** cortical TMD (upper panel) of the diluent- vs. anti-CD20-treated 14-week old female mice measured using μCT. Values are mean ± SEM (summary of two separate experiments). *n* = 17–21 mice in each group. **(D)** Representative μCT images of the data presented in **(C)** where voxel-based mineral density values are color coded as depicted in the inserted scale.

μCT analysis of mice femurs (at 14 weeks of age) revealed that B cell depletion by anti-mCD20 led to a significant 7.7% increase in the volumetric bone mineral density (vBMD) of the whole femur as compared to diluent-injected controls (*p* < 0.01, Figure 4A). This was accompanied by a 5.1% increase in the cortical tissue mineral density (TMD) and 5.2% increase in the metaphyseal TMD (*p* < 0.03, Figures 4B–D), indicating increased mineral content in the trabecular bone of the anti-mCD20-treated animals. Of note, we did not find a significant change in the trabecular vBMD (data not shown).

### Depletion of Mature B Cells Results in a Decrease in Osteoclastogenic Signals

**Figure 5:**
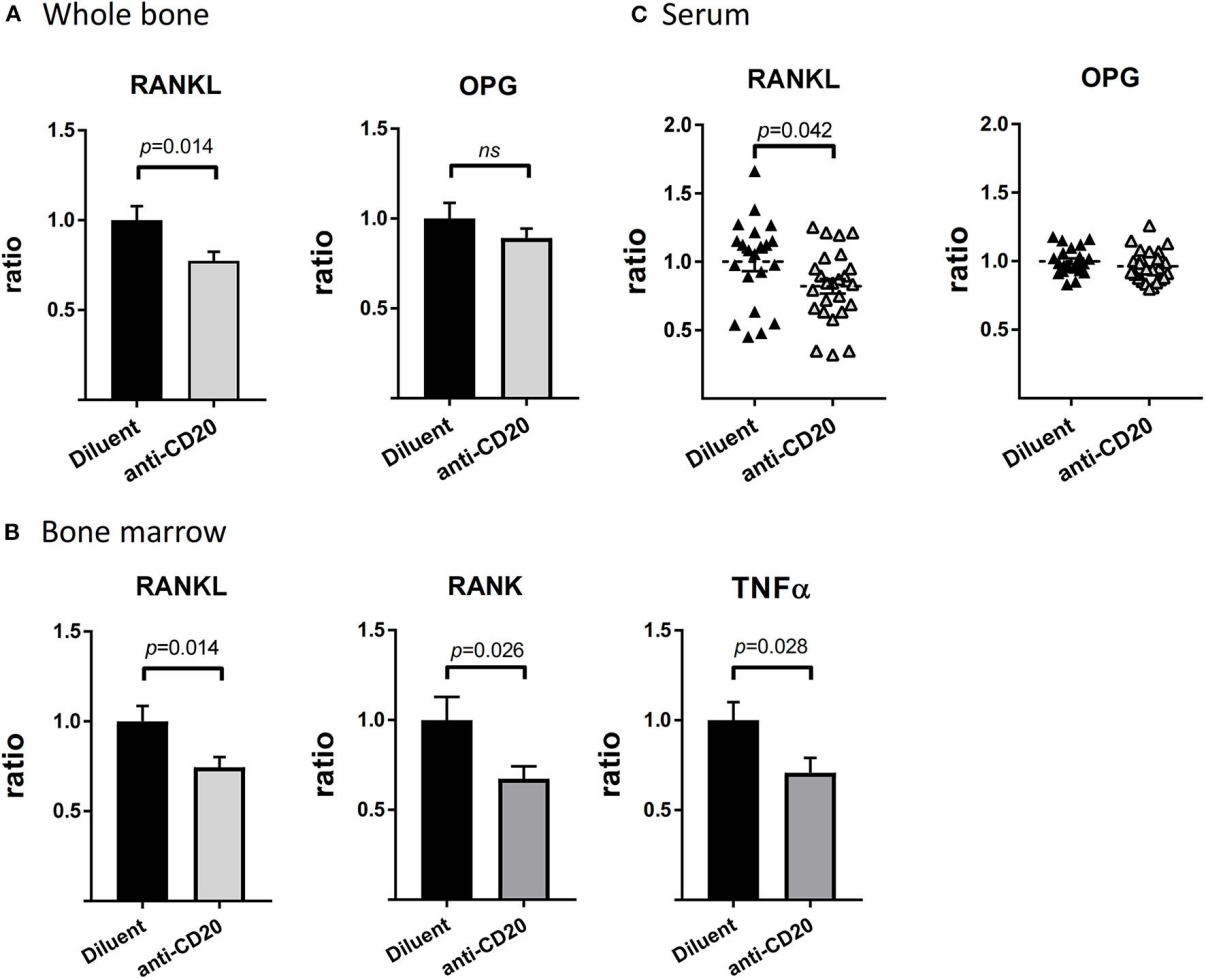
Depletion of mature B cells results in a widespread reduction of osteoclastogenic signals. **(A)** RANKL (left) and OPG (right) expression in whole bone (proximal tibia). **(B)** RANKL (left), RANK (middle), and TNFα (right) expression in the bone marrow (BM, flushed out from tibia) from diluent- vs. anti-CD20-treated 12–14-week old female mice. Values are mean ± SEM of ΔΔCT values normalized to the diluent group of each individual experiment (summary of three separate experiments with *n* = 27–31 mice in each group). Of note the levels of RANKL in the whole bone were approximately 23-fold higher than those measured in the BM. **(C)** Levels of RANKL and OPG in the sera of mice described in **(A,B)** as assessed by the ELISA method. Values are RANKL or OPG concentrations (in pg/mL) normalized to the diluent group of each individual experiment (summary of three separate experiments). The interquartile range of the RANKL levels was 68.3–109.2 pg/mL, while that of OPG was 1809–2322 pg/mL (both groups). ns = not significant.

Treatment of mice with anti-mCD20 resulted in a significant 22.5 and 25.5% decrease in the expression of RANKL, in whole bone and in the BM, respectively (*p* < 0.05), with no change in expression of OPG (Figures 5A,B). In fact, we detected no expression of OPG in the BM of either control or B cell-depleted mice. In agreement with the decreased mRNA expression of RANKL described above, we found significantly lower levels of RANKL in the serum of the B-cell-depleted mice (18% relative reduction, *p* < 0.05). Again, there was no change in the levels of OPG (Figure 5C). In addition, there was a 33% decrease in the levels of RANK, the cognate receptor of RANKL, and a 29% reduction in the expression of tumor necrosis factor α (TNFα), a potent stimulator of osteoclastogenesis (31, 32), in murine BM following treatment with anti-mCD20 (*p* < 0.05 for both parameters, Figure 5B).

### Anti-CD20-Mediated B Cell Depletion Decreases the Frequency of Total BM, as Well as B-Cell-Derived Preosteoclasts

**Figure 6:**
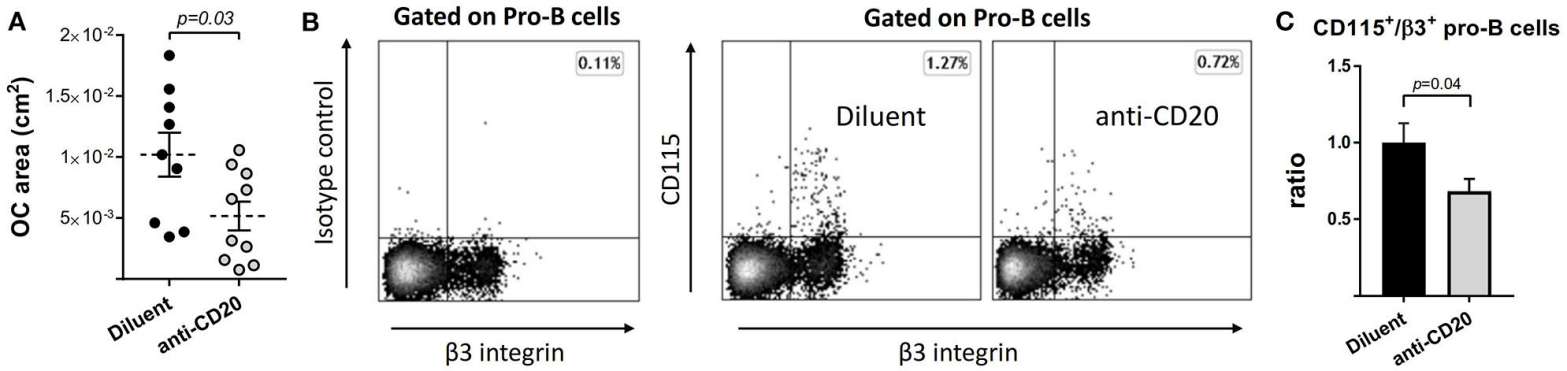
The number of B-cell-derived preosteoclasts declines following anti-CD20 treatment. **(A)** Total area of osteoclasts (OC area) grown with M-CSF and RANKL *in vitro* from bone marrow (BM) non-adherent cells isolated from mice treated with either diluent or anti-CD20. Dashed lines represent the mean ± SEM. **(B)** B-cell-derived preosteoclasts, defined as CD115^+^/β3^+^ Pro-B cells, in the BM of mice described in **(A)**. Isotype control staining is presented on the left. **(C)** Bar graphs summarizing the data presented in **(B)**. Data are mean ± SEM of values normalized to the diluent group of each individual experiment. *n* = 20 and 24 mice in the diluent- and anti-CD20 treatment groups, respectively.

In order to assess whether the decrease in osteoclastogenic signals following anti-CD20 treatment is accompanied by a decrease in the differentiation potential of the osteoclast precursors in the BM, we subjected total BM cells from either diluent or anti-mCD20-treated mice to a standard *in vitro* osteoclastogenic assay. Despite a similar proportion of CD115^+^ BM cells (13.1 ± 0.74% vs. 13.7 ± 0.82%), we found a lower osteoclastic output in the B-cell-depleted vs. control mice, as reflected by a significantly lower total calculated area of TRAP^+^ osteoclasts in the anti-mCD20-treated mice (0.0052 ± 0.0012 cm^2^ vs. 0.010 ± 0.0018 cm^2^, respectively, *p* < 0.05, Figure 6A). This finding is in line with the lower expression of RANKL (Figure 5B). We also found a lower frequency of B-cell-derived preosteoclasts (Figures 6B,C), defined as CD115^+^/β3 integrin^+^ Pro-B cells (23), which correlates with lower osteoclastogenic potential observed following B cell depletion (Figure 6A).

## Discussion

This study describes the effect of anti-CD20 antibody treatment on bone mass in hematological patients as well as in mice. Despite previous studies suggesting that administration of anti-CD20 antibodies to SLE and RA patients may lead to bone loss (8, 9), our data, albeit in a limited number of patients, suggests that this is not necessarily the case in hematological patients. In fact, treatment with anti-CD20 antibodies was associated with preservation of bone mass in patients with follicular lymphoma receiving rituximab maintenance (Figure 2). The difference in the bone outcome in lymphoma patients, compared to certain cohorts of patients with rheumatic diseases, may stem from the chronic inflammatory nature of the latter where immune cells other than B lymphocytes are involved, and the fact that the use of anti-CD20 antibodies in a rheumatological clinical setting may be reserved for more severe cases, that is, patients who failed other disease-modifying anti-rheumatic agents (DMARDs) (33).

A similar CT-based method for assessing BMD was also used by Svendsen et al. (34) to assess the skeletal effects of RCHOP immunochemotherapy among patients with diffuse large cell lymphoma. In line with their findings, a subgroup analysis of the control patients (no maintenance) treated with RCHOP regimen (Table 1) indeed demonstrated a significant decrease in bone mass over two consecutive scans (data not shown).

The human data has a clear advantage of being the most clinically relevant. However, it is also inherently masked by confounding factors, such as chemotherapy and glucocorticoid use during the induction phase. For this reason, we opted to monitor bone mass during the maintenance phase, which is devoid of treatments other than anti-CD20.

Conceptually, the bone preserving effect of anti-CD20 in lymphoma patients could either derive from a better control of the disease at the microscopic level, that is, lower levels of minimal residual disease (MRD) (could not be evaluated in this study) and/or a direct effect of anti-CD20 on bone metabolism.

Since we could not explore the skeletal effect of anti-CD20 in healthy individuals, we employed a murine model (wild-type mice). This model has the advantage of being free of confounding factors such as the disease course, and in particular, the lymphoma itself and lymphoma stage that could potentially affect bone remodeling. As shown in Figure 4, anti-mCD20 treated mice displayed a modest but significant increase in bone mass, which supports the premise that depletion of mature B cells by itself plays a role in bone remodeling. Our data also suggest that anti-CD20-induced B cell depletion reduces the osteoclastogenic output originating from bone cells as well as other BM cells. B cells express RANKL, the master regulator of osteoclastogenesis (2, 35). We found decreased expression of RANKL in whole bone and BM of anti-mCD20-treated mice, which was in agreement with the lower serum levels of RANKL but no alteration in the serum levels of OPG. However, we believe that the downregulation of RANKL cannot be solely explained by the partial depletion of IgM^+^ B cell in the BM due to the much higher expression of RANKL in whole bone (bone + BM) relative to that in the BM. Therefore, the 25% reduction in the RANKL expression in the whole bone probably results from a reduced expression of RANKL in bone cells, i.e., osteoblasts and/or osteocytes (36, 37). The mechanism by which B cells affect RANKL expression in bone cells remains to be determined. Be that as it may, our findings suggest that CD20-directed therapy has a significant influence on bone remodeling, as evidenced by the bone-preserving effect of rituximab observed in our cohort of patients with FL. It is also possible that the favorable skeletal effects of rituximab maintenance offsets a potentially deleterious effect induced by glucocorticoids included in some of the more commonly used induction regimens, for example, RCHOP/RCVP (24).

Although the number of preosteoclasts (defined as CD115^+^ BM cells) did not change following treatment with anti-mCD20, however, the expression of RANK, the cognate receptor for RANKL, was significantly reduced. According to studies in human patients, there is a direct correlation between the expression of RANK and the osteoclastogenic potential of BM cells *in vitro* (38). Indeed, BM cells from the B-cell-depleted mice in our model had lower osteoclastogenic capacity *ex vivo* (Figure 6A). We also suggest that the lower osteoclastogenic capacity may be partially explained by a smaller population of B-cell-derived osteoclast precursors defined as CD115^+^/β3 integrin^+^ Pro-B cells (Figures 6B,C). In addition, the reduced TNFα expression in BM cells of anti-mCD20-injected mice (Figure 5B) is consistent with the data of Toubi et al. demonstrating lower secretion of this important cytokine by human monocyte-derived macrophages (HMDMs) isolated from rituximab-treated RA patients (39).

Since IL-7 was shown to induce bone loss (20), we analyzed the levels of this cytokine following anti-CD20-mediated B cell depletion. Interestingly, the levels of IL-7 did not differ between control and anti-mCD20-treated mice. This is somewhat counterintuitive considering the fact that IL-7 acts as a growth factor for the B cell lineage (40). In addition, it was previously suggested that depletion of mature B cells “rejuvenates” B cell lymphopoiesis, a phenomenon that is significantly more conspicuous in old mice, leading to significant changes in the frequencies of Pro/Pre-B cells fractions (41, 42). Nevertheless, the unchanged levels of IL-7 mRNA in our study are in line with our flow cytometry data (Figure 3C) and support the work by Martin et al. (43), who reported that regulation of IL-7 expression does not involve feedback loops. Rather, the availability and the abundance of IL-7 is determined by the amount of IL-7R-bearing hematopoietic cells, for example, Pro- and Pre-B cells, but not IgM^+^ B cells, that merely serve as a “sponge” for IL-7 molecules. Plausibly, feedback regulation of B lymphopoiesis elicited by alterations in the peripheral (mature) B cell pool, does exist, as also suggested by Shahaf et al. (44), but it does not seem to involve IL-7.

Li et al. (10) reported that transgenic mice lacking IgM^+^ B cells due to an inability to form μ heavy chains (the μMT/μMT mouse model) had a low bone mass phenotype that was largely determined by diminished levels of OPG, for which mature B cells are a substantial source. We assume that the discrepancy in the bone phenotypes between our data and that of Li et al. (10) may be explained primarily by a different experimental setting, that is, acquired vs. congenital depletion of mature B cells. Mechanistically, we could not detect OPG transcripts by RQ-PCR in splenocytes, BM cells or isolated BM B cells, even in high amounts of cDNA (data not shown).

One limitation of this study is that we used only female mice for anti-CD20 injections. Notably, previous reports on the effect of B cells and the B-cell-expressed RANKL on bone remodeling were performed in female mice, primarily because of the relevance to postmenopausal osteoporosis (11, 45, 46) and the results indicated that B cells mediate, at least in part, the effect of estrogen on bone mass. In light of these important reports, we selected female mice for our study. Although it is reasonable to assume that males will respond similarly (as most of testosterone is aromatized to estrogens), the gender effect is still an important issue that merits further investigation. Clinic-wise, considering the age range of the current patient cohort, osteoporosis is significantly more common in women, further supporting our choice of the female murine model.

**Figure 7:**
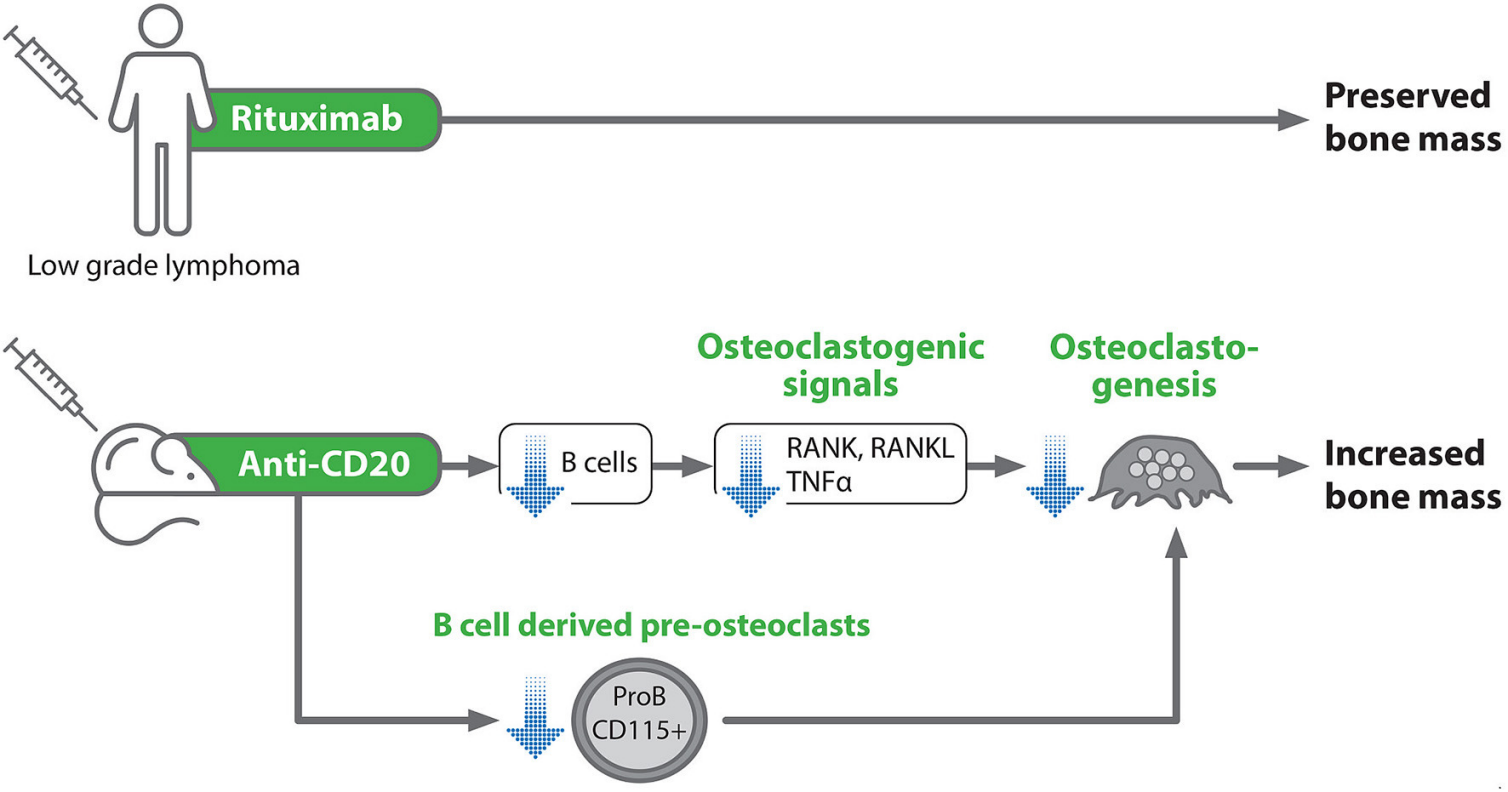
Schematic representation of the experimental design and summary of the main study findings. Blue arrows denote an inhibitory effect.

In summary, we show here for the first time that rituximab treatment in lymphoma patients is not associated with deleterious skeletal effects, at least in the setting of maintenance therapy. It should also be stressed that it is extremely challenging nowadays to design retrospective or prospective clinical studies to explore the skeletal effects of anti-CD20 antibodies in lymphoma patients and probably other clinical settings, due to the absence of an adequate control group, that is, a group of patients not receiving anti-CD20. Thus, the method used in this study for assessing bone mineral density is feasible and can be implemented for further research in the field. Moreover, our mouse model data suggest that acute B cell depletion may directly affect bone metabolism by downregulating osteoclastogenic signals including RANKL, resulting in increased bone mass (Figure 7).

## Data Availability Statement

The raw data supporting the conclusions of this article will be made available by the authors, without undue reservation, to any qualified researcher.

## Ethics Statement

The studies involving human participants were reviewed and approved by the Sourasky Medical Center Institutional Ethical Committee, permit number 0418-18-TLV. Written informed consent for participation was not required for this study in accordance with the national legislation and the institutional requirements. The animal study was reviewed and approved by Institutional Animal Care and Use Committee of the Tel-Aviv University (permit number: 01-016-010).

## Author Contributions

AK designed and performed the experiments, analyzed the data, and wrote the manuscript. IK collected and analyzed patient data. NS and CP allocated patients for the study and provided input on the data analysis. DN and MM designed the study, co-directed the overall project, analyzed the data, and wrote the paper. YG designed the mice study, analyzed the data, and wrote the paper. HSO analyzed the data, provided critical input, and wrote the paper. AG, SH-B, TL, NB-C, ZA, and MI performed experiments and discussed the results. All authors contributed to the article and approved the submitted version.

## Conflict of Interest

The authors declare that the research was conducted in the absence of any commercial or financial relationships that could be construed as a potential conflict of interest.
