# Supplementary figures and images for: Anti-CD20-Mediated B Cell Depletion Is Associated With Bone Preservation in Lymphoma Patients and Bone Mass Increase in Mice
Source: Front Immunol. 2020 Oct 19;11:561294. doi: 10.3389/fimmu.2020.561294 (PMC7604358; doi:10.3389/fimmu.2020.561294)

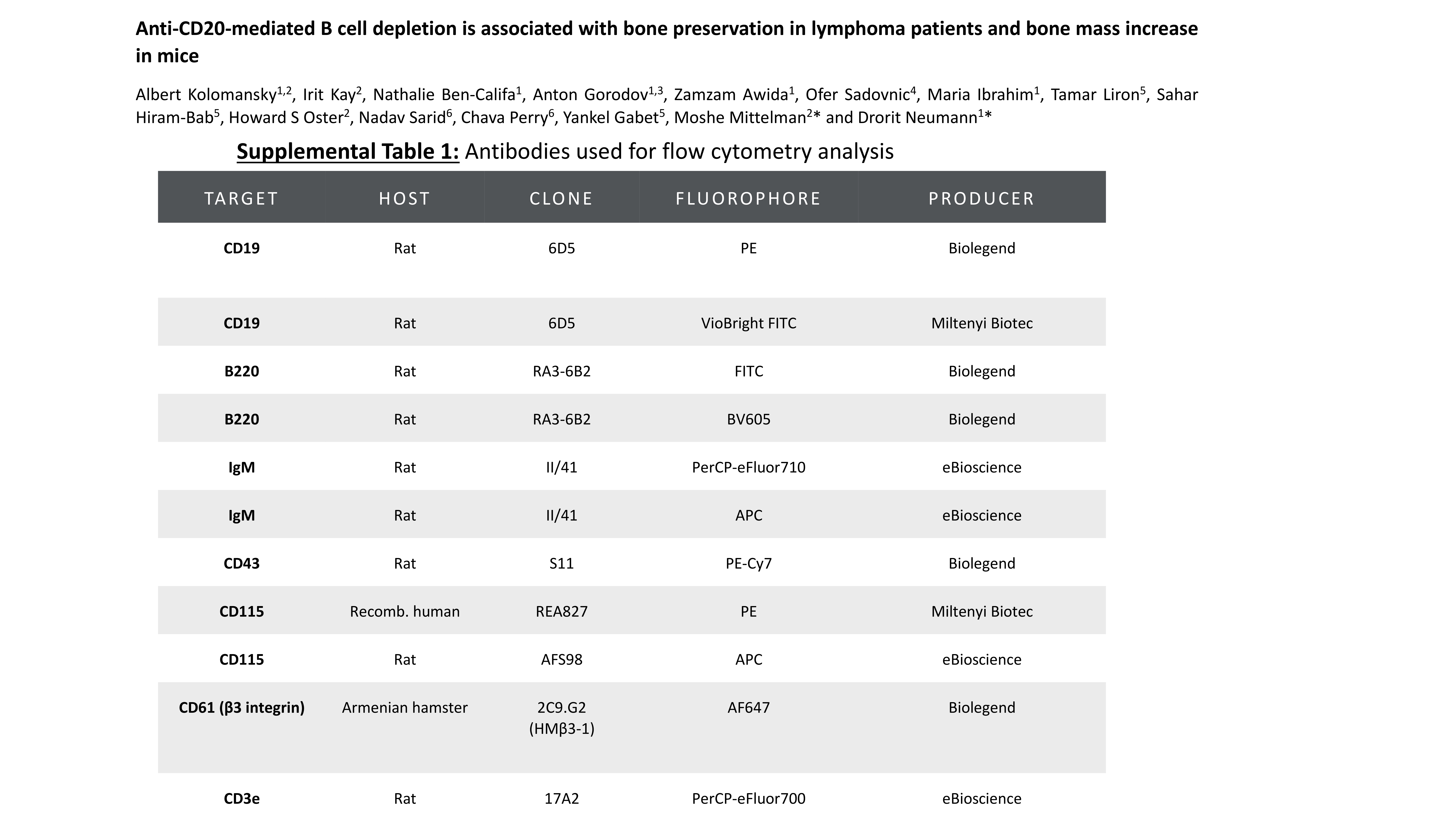

Supplement: Supplementary file 1 [file Image_1.JPEG]
